# Optimization of Tetracycline Sonophotocatalytic Degradation by Cerium-Based Metal–Organic Framework

**DOI:** 10.1021/acsomega.6c02738

**Published:** 2026-05-18

**Authors:** Hanieh Shahabinejad, Mojtaba Binazadeh, Feridun Esmaeilzadeh, Reza Andalibi

**Affiliations:** † Department of Chemical Engineering, School of Chemical and Petroleum Engineering, 37551 Shiraz University, Shiraz 7134851154, Iran; ‡ School of Engineering, 4396 Lancaster University, Bailrigg LA1 4YW, United Kingdom

## Abstract

The increasing consumption of pharmaceuticals, particularly antibiotics, and their discharge in used water adversely impact wastewater treatment facilities, posing a significant environmental challenge. This study investigates the removal of tetracycline from aqueous media using a novel cerium-based metal–organic framework synthesized through sonochemical techniques. The sonophotocatalyst was characterized by using infrared spectroscopy, X-ray diffraction analysis, and scanning electron microscopy. Response surface methodology was employed to optimize tetracycline removal conditions across five levels of photocatalyst loading, initial tetracycline concentration, contact time, and pH. Optimal removal conditions were achieved at pH 7, an initial tetracycline concentration of 145 ppm, a photocatalyst loading of 1307 ppm, and a contact time of 111 min, yielding an 89% removal efficiency. The synthesized Ce-MOF demonstrated stability and sustained sonophotocatalytic activity, presenting a promising solution for pharmaceutical contamination in advanced water-treatment applications.

## Graphical abstract

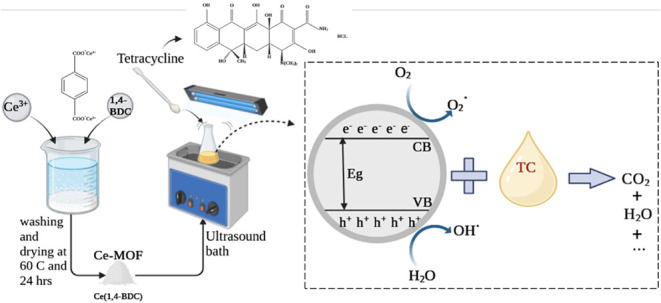

## 1 Introduction

According to the World Health Organization (WHO), pharmaceutical contaminants are routinely detected in water sources, sewage, and drinking water. The concentration of antibiotics ranges from ng L^–1^ to μg L^–1^ in untreated household wastewater and 100–500 mg L^–1^ in pharmaceutical/hospital wastewater.^1^ Antibiotics significantly impact water treatment processes.^2^ Pharmaceutical contamination originates from pharmaceutical company and hospital effluents, excretion of unabsorbed drugs through human/animal waste, and improper disposal of expired or unused medications.^3^ The water solubility, persistence, and toxicity of antibiotics have made their presence in aquatic environments a critical environmental concern.^4,5^ There is a direct correlation between antibiotic usage and bacterial resistance.^6^ The most significant adverse effect of antibiotic discharge into the environment is genetic mutation in microorganisms, leading to antibiotic resistance that threatens both human/animal health and environmental stability.^7^ Tetracyclines (TCs) represent a widely prescribed class of antibiotics.^8^ These compounds have been detected in surface waters,^9^ groundwater,^10^ and soils,^11^ with their ecological hazards and potential toxicities well-documented.^12–14^ Conventional wastewater treatment plants exhibit varying TC removal efficiencies, ranging from 12%^15^ to 80%,^16^ highlighting their limitations in achieving complete micropollutant elimination.

Various methods have been employed for TC removal, including biological processes,^17^ membrane treatment,^18^ ion-exchange resins,^19^ ozonation,^20^ disinfection,^21^ electrochemical methods,^22^ immobilization,^23^ adsorption,^24^ and advanced oxidation processes (AOPs).^25^ Among these, adsorption stands out due to its simplicity, cost-effectiveness, efficiency, and the recyclability of adsorbents.^26,27^ Photocatalysis, on the other hand, is recognized for its environmental safety, high efficiency, and cost-effectiveness, making it a widely adopted approach.^28^ As an AOP, photocatalytic decontamination can degrade TCs into nonhazardous products such as H_2_O, CO_2_, and NH_3_.^28,29^ Some photocatalysts also exhibit significant pollutant adsorption capabilities.^30^ CoP-3 and Zn@Co–N–C-1000/PMS MOF are among the reported catalysts employed for TC degradation. Although their application results in high TC degradation, their utilization has drawbacks. CoP-3 is synthesized with toxic dimethylformamide (DMF), dried at 160 °C, utilizes a chemical oxidant (peroxymonosulfate, PMS) for TC degradation, and operates at ≤20 ppm tetracycline (TC).^31^ Zn@Co–N–C-1000 also requires pyrolysis at 1000 °C and uses PMS, having been tested at 50 ppm TC.^14^

Metal–organic frameworks (MOFs) are among the most effective photocatalytic adsorbents.^30^ These crystalline materials consist of self-assembled metal ions or clusters coordinated with organic ligands. Although MOF-type structures were first discovered in the 1960s, the term “MOF” was formally introduced by Yaghi et al. in the 1990s.^32^ MOFs are crystalline, porous materials whose advantages include exceptionally high surface areas and adjustable pore dimensions, which facilitate superior gas uptake, selective adsorption, and molecular sieving.^33^ Their modular nature permits deliberate chemical modification, tailoring them for specific roles in catalysis, chemical sensing, and controlled release.^34^ When employed as photocatalysts, MOFs leverage their inherent porosity to maximize light harvesting and substrate accessibility, while the strategic choice of metal clusters and organic linkers allows fine-tuning of their electronic structure for visible-light activation.^35^ Innovations such as defect engineering and composite formation have recently enhanced their charge-carrier dynamics and operational durability, positioning MOF-based photocatalysts as viable, eco-friendly options for degrading persistent contaminants.^36^ Limited studies have explored TC elimination using various MOFs, including ZIF-8,^37^ PCN-128Y,^38^ NH_2_-MIL-101,^39^ MOF-5,^40^ alginate-GO-ZIF67,^41^ MIL-68­(Al)/GO pellets,^42^ and multiwalled carbon nanotubes loaded on MIL-53­(Fe).^43^ MOFs facilitate both adsorption and photocatalytic decomposition.^44,45^ Their first photocatalytic application was reported in 2005 for dye removal.^46^ Tables 1 and 2 summarize the previous research in this area.

**Table 1:** Adsorption of TC via MOFs-Based Absorbent

Material	Adsorbent Dosage(g/L)	t^a^ (min)	pH	T^b^ (K)	Qmax(mg/g)	Surface areas(m2/g)	Pore size (nm)	Ref.
Cu-TCPP	0.2	120	9	R	150	342.72	0.256	^47^
PCN-128Y	0.1	30		298	723.93	1139.20	1.13	^38^
NH_2_-MIL-101(Fe)	0.03	90			93.05	403.55	2.43	^48^
NH_2_-MIL-101	0.1	210	7		378			^39^
MIL-53(Fe)	0.2	600		298	247.7	52.18	8.81	^49^
alginate-GO-ZIF67	0.5	720	4		456.62	138.62	15.43	^41^
nZVI/MIL-101(Cr) (nZVI = nano zerovalent iron)	0.1	480		318	625	786.4	2.43	^50^
UiO-67/NSC (NSC = N,S codoped carbon dots)	0.02	180	3	318	427.35			^51^
H-UiO-66–17.3 nm	1.0	720	5	303	666.67	1168.10	17.2	^52^
MOFs-derived N doping-C	0.2	240	6		330.98	2402.53	3.33	^53^

a Equilibrium time.

b Adsorption temperature.

**Table 2:** Degradation of TC via MOFs-Based Photocatalyst

Catalyst	Antibiotic dosage(mg/L)	Degradation efficiency (%)	Catalyst dosage(g/L)	pH	Fenton reagent	Dark reaction time (min)	Illumination time(min)	Recycle	Ref.
MIL-101	50	82.52	0.15	10.2	10 mL/L H_2_O_2_	30	20	3	^54^
Fe-MIL-101	50	96.6	0.5	-	-	60	180	3	^42^
Cu-TCPP	10	86.3	0.05	9	1% H_2_O_2_	60	360	-	^47^
perylene imide-modified NH_2_-UiO-66	10	88	0.2	9.3	-	30	100	3	^55^
ZIF-8@TiO_2_	100	92	0.6	-	-	30	120	5	^56^
CuBi_2_O_4_@ZIF-8	20	75.3	0.5	-	-	60	60	5	^51^
Ce-MOF	145.5	88.8	1.3	7.	-	60	111	6	This work

Existing research on antibiotic adsorption and photodegradation using MOFs primarily focuses on material characterization rather than removal efficiency. Consequently, there is significant potential for exploring the removal of antibiotics using MOF-based photocatalysts. For example, the synergistic enhancement of photocatalytic activity via simultaneous ultrasonic irradiation (sonophotocatalysis) remains an underexplored area warranting further investigation.^57–59^ Recent work on MIL-88B has demonstrated improved degradation efficiency through the combined effects of ultrasound and light irradiation, highlighting the potential of this integrated approach.^60^

In previous work, a novel Ce-MOF was sonochemically synthesized, offering several distinct advantages. The synthesis (i) employs water as a benign solvent instead of toxic organic media; (ii) proceeds at room temperature, rendering the process low-energy, low-cost, and safe; (iii) utilizes inexpensive and readily available raw materials alongside common laboratory equipment, making the approach simple and attractive for practical applications;^61^ and (iv) eliminates doping which simplifies the synthesis process and reduces costs. This research optimizes sonophotocatalytic TC degradation via Ce-MOF by varying the pH, initial TC concentration, catalyst dose, and process time. Optimal conditions are determined using Design Expert software with the central composite method. The findings from this study can provide valuable insights into future modeling and scaling of pharmaceutical, municipal, and hospital wastewater treatment facilities.

## 2 Experimental Section

### 2.1 Materials

Cerium nitrate, benzene-1,4-dicarboxylate (terephthalic acid), ethanol, hydrochloric acid, and sodium carbonate were purchased from Merck. Analytical-grade tetracycline hydrochloride was generously provided by Sinadarou Laboratories Company. Deionized water was used as the solvent.

### 2.2 Sonochemical Synthesis of Ce-MOF

An appropriate amount of benzene-1,4-dicarboxylate was added to 50 mL of deionized water under continuous stirring to achieve a final concentration of 8.4 mmol/L. Ammonia solution (46 wt.%) was gradually added to the solution to adjust the pH to 7. An ultrasonic probe (QSONICA, Q700) was then immersed in the solution under ambient conditions. Subsequently, 10 mL of Ce­(NO_3_)_3_ aqueous solution was gradually added to the terephthalic acid solution to achieve a final molar ratio of benzene-1,4-dicarboxylate to Ce­(NO_3_)_3_ equal to 1.5. The sonication process was carried out at a power output of 50% amplitude for 60 min. The resulting mixture was placed on a stirrer for 12 h to ensure uniform mixing. Finally, the precipitate was collected, washed, and dried at 60 °C for 24 h.^61^

### 2.3 Ce-MOF Characterization

Fourier transform infrared (FTIR) spectroscopy analysis was performed over the range of 400–4000 cm^–1^. X-ray diffraction analysis was conducted using a Bruker Tensor II and a Bruker D8 Advance diffractometer with graphite-monochromated Cu Kα radiation (λ = 1.54056 Å) at 25 °C. The scanning rate was set at 0.02° min^–1^ across a 2θ range of 10–80°. The sample morphology was characterized using transmission electron microscopy (TEM, Philips EM 208S) and field emission scanning electron microscopy (FESEM, TESCAN MIRA III) with an acceleration voltage of 100 keV.

### 2.4 TC Adsorption Experiments

For the TC adsorption experiments, Ce-MOF was dispersed in 30 mL of tetracycline solution (210 ppm) at pH 6 to achieve a final Ce-MOF concentration of 1000 ppm. The adsorption studies were conducted under dark conditions in a 50 mL glass beaker. Kinetic adsorption experiments were performed at room temperature using two different mixing methods: magnetic stirring and sonication. In the stirring experiment, the solution was continuously mixed at 4000 rpm using a magnetic stirrer, while in the sonication experiment, the beaker was placed in an ultrasound bath operating at 50 W. At 30-min intervals, 3 mL aliquots were withdrawn and filtered through a 0.22 μm PTFE syringe filter to remove the Ce-MOF particles. The TC concentration in the filtered solution was determined using UV–vis spectroscopy (Unico S-2150 spectrophotometer) at the maximum extinction wavelength (λ_max_) of 360 nm, applying Beer’s law for concentration calculations.

Adsorption equilibrium studies were conducted under stirring conditions at the optimal point of the photocatalytic reaction. Samples were collected at predetermined intervals, separated by centrifugation and filtration, and analyzed using UV–vis spectroscopy to determine the remaining TC concentration. The adsorption capacity of Ce-MOF was calculated using eq 1, while the removal efficiency was determined using eq 2

1 $$\mathrm{Adsorption capacity}=\frac{\left(C_{0}-C_{e}\right)V}{m}$$

2 $$\mathrm{Removal efficiency}\%=\frac{\left(C_{0}-C_{e}\right)}{C_{0}}\times 100$$

Here, the initial and equilibrium liquid phase concentrations of TC are given by C_0_ and C_e_ respectively (both in mg·L^–1^), V denotes the total solution volume (in L), and m represents the adsorbent mass (in g).

### 2.5 TC Degradation Experiments

The TC degradation experiments using Ce-MOF were conducted in a batch sonophotocatalytic reactor, as illustrated in Figure 1. The irradiation source consisted of a UV-A lamp (OSRAM ULTRA-MED FDA 400W) positioned 10 cm above the reaction solution. The UV intensity, measured by using a UV radiometer (UVA 365 Lutron), was approximately 1.8 mW/cm^2^. Solution pH adjustments were made by using either 0.1 M HCl or 0.1 M CaCO_3_. The reaction vessel was placed in an ultrasonic bath (50 W) to enhance solution mixing and aeration. To mitigate the heating effects of the UV lamp, we continuously circulated air through the chamber as a cooling agent. The ultrasonic bath temperature was maintained at 298 K through water circulation.

**Figure 1:**
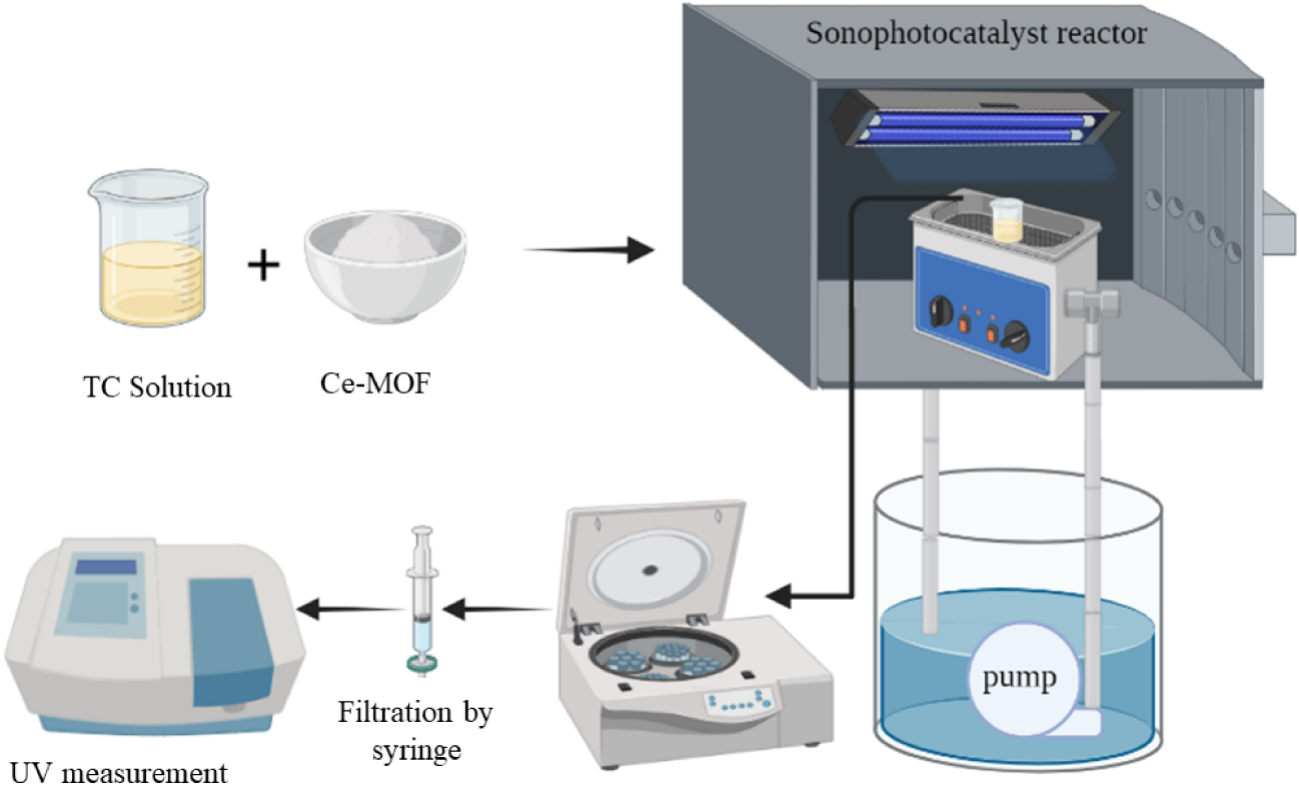
Schematic representation of the approach and the setup for sonophotocatalytic degradation of TC.

The effects of several parameters on photocatalytic TC degradation were investigated, including the Ce-MOF concentration, TC concentration, pH, and reaction time. Prior to photocatalytic testing, the TC adsorption equilibrium on the Ce-MOF photocatalyst was established by maintaining the dispersion under dark conditions for 60 min. During the photocatalytic experiments, samples of less than 3 mL were withdrawn for UV–vis analysis.

To evaluate the role of visible light in TC degradation, control experiments were conducted under visible light illumination while maintaining all other experimental conditions constant. The degradation efficiency was calculated using eq 3

3 $$\%\mathrm{Degradation efficiency}=\frac{\left(C_{i}-C_{f}\right)}{C_{i}}\times 100$$

where C_i_ represents the initial TC concentration (mg·L^–1^) and C_f_ denotes the final TC concentration (mg·L^–1^). For stability and reusability assessment, the solid catalyst was recovered after each reaction cycle, washed thoroughly with deionized water and ethanol, and dried at 60 °C.

## 3 Experimental Design and Optimization by Response Surface Methodology

The selection of screening parameters is fundamental for effective process modeling and optimization.^62–66^ A systematic experimental approach, incorporating carefully designed parameter ranges, enables a thorough analysis of response variance and facilitates the development of an optimized model. This study employs the central composite design (CCD) within the response surface methodology (RSM) framework to evaluate the influence of various parameters on the photocatalytic degradation efficiency.

The experimental design, detailed in Table 3, encompasses four key parameters with specific ranges: Ce-MOF concentration (500–1500 ppm), TC concentration (85–225 ppm), pH (4–10), and reaction time (35–180 min). Through numerical modeling, the interplay of these parameters was systematically analyzed to determine optimal conditions for TC degradation.

**Table 3:** Independent Process Variables in Degradation of Tetracycline by Ce-MOF and the Levels Investigated following a Central Composite Design

Process Variable	Unit	Minimum (−2)	Low (−1)	Mean (0)	High (1)	Maximum (2)
TC concentration	ppm	85	120	155	190	225
Catalyst loading	ppm	500	1000	1500	2000	2500
Time	min	0	45	90	135	180
pH	-	4	5.5	7	8.5	10

## 4 Results and Discussion

### 4.1 Characterization

#### 4.1.1 Fourier-Transform Infrared Spectroscopy (FT-IR)

The functional groups present in Ce-MOF were characterized using FTIR analysis. Figure 2a (pattern before degradation) presents the FTIR spectrum of fresh Ce-MOFs synthesized via the sonochemical method. The spectrum reveals several characteristic peaks: Ce–O stretching vibration at approximately 511 cm^–1^,^67^ substituted groups in ortho, meta, and para positions of the benzene ring in the 730–770 cm^–1^ region, and C–O bond stretching vibrations within 1000–1200 cm^–1^.^68^ Additional peaks were observed at 1400–1540 cm^–1^ (O–H bending), 1550–1600 cm^–1^ (C=O stretching and carboxylate anions), and 1600–1700 cm^–1^ (C=C stretching). The spectrum also shows aromatic C–H stretching near 2900–3000 cm^–1^, and OH groups at 3400–3500 cm^–1^.^69^ The presence of ν–OH bands around 3465 cm^–1^ indicates the incorporation of water molecules as reactants in the product.^70^

**Figure 2:**
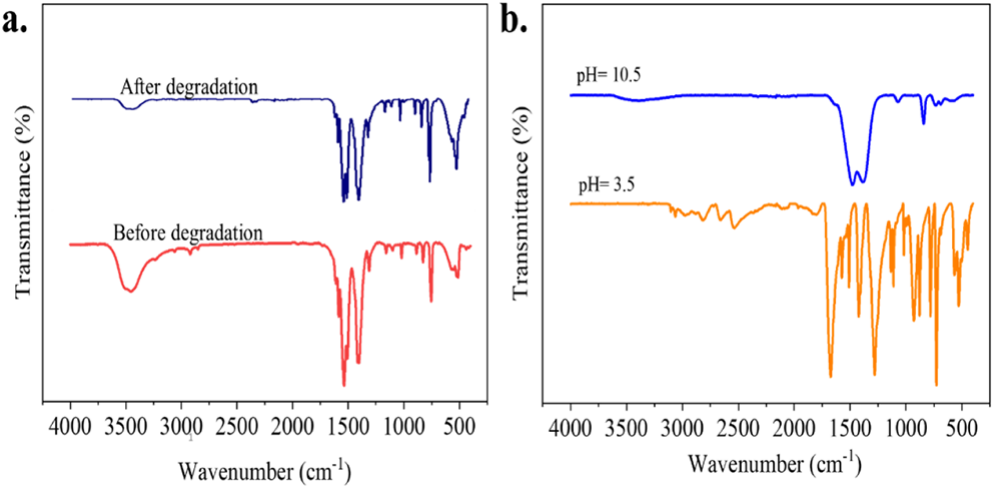
(a) FTIR spectra images of Ce-MOF before degradation and after degradation at pH 7. (b) FTIR spectra of Ce-MOFs in a stability test under acidic (pH 3.5) and basic (pH 10.5) conditions.

The selection of an appropriate pH range for photocatalytic degradation was informed by a literature review and experimental validation. Based on previous studies by Masoumi et al. (pH 2–11)^56^ and other TC removal investigations,^71–75^ combined with our preliminary TC removal tests (see Figure 6a below), a pH range of 4–10 was identified. To verify the stability limits of the Ce-MOF, additional experiments were conducted at pH 3 and 11, which resulted in the complete dissolution of the MOF structure. Further stability tests at pH 3.5 and 10.5 were performed using 500 ppm MOF suspensions. After 10 min of stirring, the samples were centrifuged, separated, and dried at 60 °C for 24 h. FTIR analysis of these samples (Figure 2b) revealed the degradation of certain bonds, confirming structural instability at these pH values. Consequently, all TC degradation experiments were confined to the pH range of 4–10 to maintain Ce-MOF structural integrity.

To determine the stability of the MOF after being used for TC decomposition, FTIR spectra were collected from the used Ce-MOF after drying in an oven at 60 °C for 24 h (Figure 2a pattern after degradation). Given that the spectra are almost similar, we conclude that the TC decomposition reaction has not significantly affected the structure of the Ce-MOF (note that the minor damping of the transmission around 3500 cm^–1^ can be attributed to the effect of drying, which removes the trapped water molecules^76^).

#### 4.1.2 XRD of Ce-MOF

The X-ray diffraction (XRD) pattern of the Ce-MOF before and after being used in TC degradation is shown in Figure 3. The diffractogram of fresh Ce-MOF exhibits peaks at 2θ = 9.1°, 14.5°, 15.3°, 18.2°, 28.2°, 29.3°, and 30.0°.^61,68^ The XRD pattern of used Ce-MOF is presented in Figure 3. It can be readily seen from the figure that the XRD patterns of fresh and used Ce-MOF perfectly match, indicating its stability upon application.

**Figure 3:**
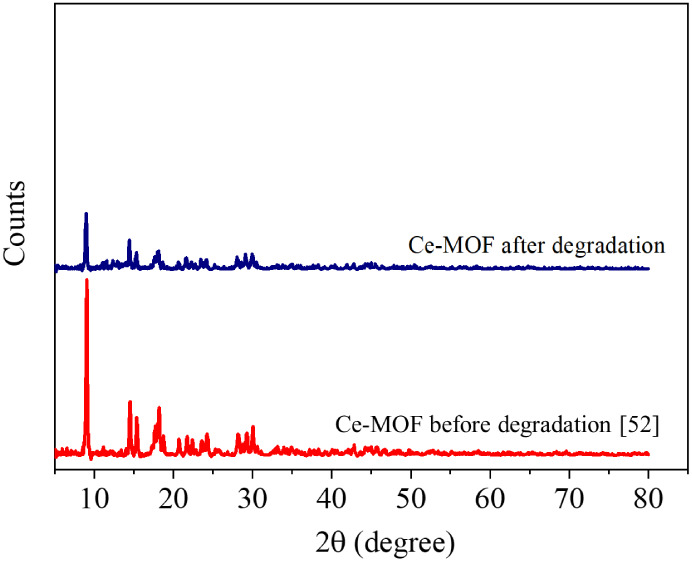
XRD pattern of Ce-based MOF before and after being used in TC degradation.

#### 4.1.3 Electron Micrographs (FE-SEM) and Energy Dispersive X-ray Spectroscopy (EDX) Characterization

The FE-SEM images of the Ce-MOF sample are shown in Figure 4a–c at three different magnifications. The micrographs show the agglomerated structure of the powder, consistent with our previous work. EDX analysis is shown in Figure 5. The elements Ce, C, and O are depicted using purple, blue, and green colors, respectively. The mapping reveals a homogeneous distribution of cerium throughout the particles, which overlaps perfectly with the carbon and oxygen signals. This spatial arrangement and uniform dispersion provide additional confirmation of the successful synthesis of the Ce-MOF and indicate that Ce is incorporated into the MOF framework as the primary metal node, rather than being deposited as a separate phase on the surface. Figure 5e displays the elemental composition within the material’s framework, showing a pure structure free from any contaminants. Quantitative EDX analysis gave an average Ce content of 31.52 wt %, confirming both the elemental composition and the uniform distribution of Ce throughout the material.

**Figure 4:**
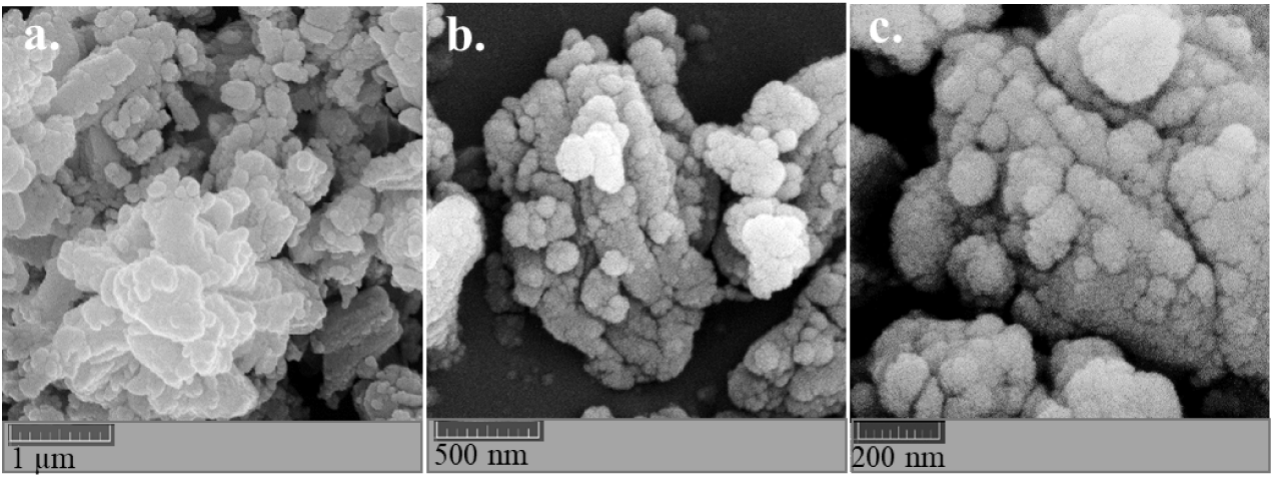
FE-SEM images of the Ce-MOF at different scales: (a) 1 μm, (b) 500 nm, and (c) 200 nm.

**Figure 5:**
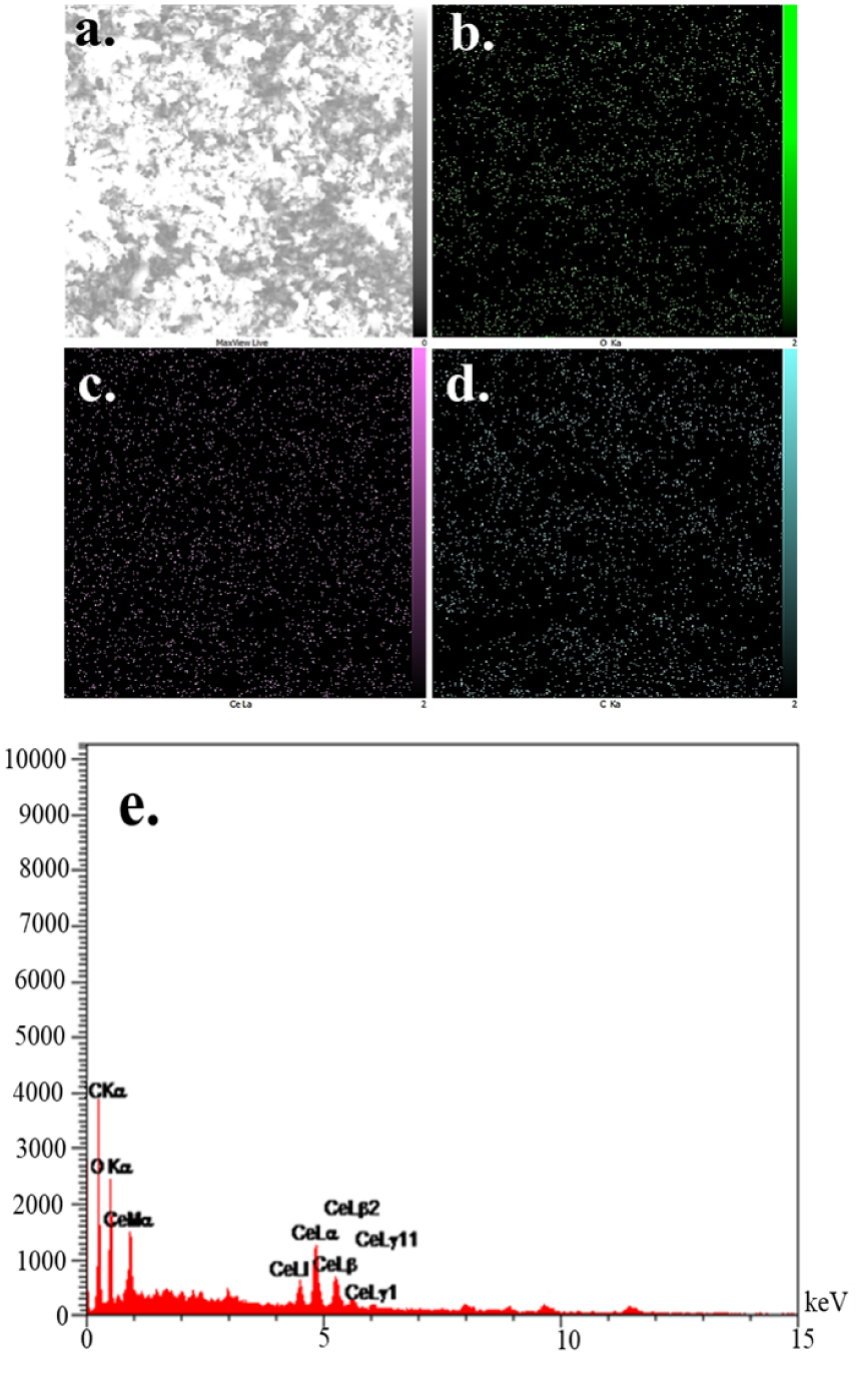
(a) SEM image of Ce-MOF and corresponding color-coded EDX spectral maps of (b) oxygen in green color; (c) cerium in purple color; (d) carbon in blue color; (e) EDX spectrum of Ce-MOF containing O, Ce, and C.

### 4.2 Effect of Sonication

The photocatalytic degradation of organic compounds occurs through the formation of hydroxyl radicals (•OH) on the photocatalyst surface during photolysis. The combination of photocatalytic and ultrasonic irradiation (sonophotocatalysis) enhances the degradation rate of organic pollutants by increasing the generation of reactive radicals. The heterogeneous catalyst provides additional nucleation sites for cavitation-induced bubble formation, thereby promoting H_2_O molecule hydrolysis and •OH formation. This creates a synergistic effect between sonolysis and photocatalysis that drives the sonophotocatalytic degradation mechanism of organic pollutants.^77^

The sonophotocatalytic approach offers several advantages in pollutant degradation.^78^ The sonolytic cleavage of water enhances the production of oxidizing species in the liquid phase. Ultrasonic irradiation reduces Ce-MOF particle aggregation, improving the mass transfer of organic pollutants to the catalyst surface. The physical effects of acoustic cavitation prevent catalyst particle aggregation in an aqueous solution, increasing the active surface area. Additionally, ultrasonic waves continuously clean the catalyst surface, preventing the accumulation of pollutants and reaction intermediates generated during degradation. During the process, antibiotic molecules adsorbed on the photocatalyst surface react with the photocatalytically generated active radicals. Simultaneously, shock waves from cavitation-induced bubbles cause the desorption of antibiotic molecules from the photocatalyst surface, which can limit photocatalytic degradation. However, radicals formed by the transient implosion of cavitation bubbles near photocatalyst particles facilitate the additional degradation of desorbed, unreacted antibiotic molecules. The efficiency of sonophotocatalysis is enhanced by smaller nanoparticle sizes and larger active surface areas.

Given the multiple parameters affecting sonophotocatalytic degradation performance,^78^ a comprehensive investigation is essential for process optimization and potential industrial application. Key parameters identified in previous studies with other photocatalysts include catalyst loading, pH, and initial antibiotic concentration.^79^ These factors primarily influence reactive radical generation, antibiotic removal efficiency from the catalyst surface, and degradation byproduct transformation. To investigate sonolysis effects on TC photocatalytic oxidation, comparative experiments were conducted under various conditions: adsorption with continuous stirring, photocatalytic oxidation, sonocatalytic oxidation, and sonophotocatalytic oxidation. All experiments utilized the Ce-MOF photocatalyst under standardized conditions (catalyst loading: 1000 ppm, initial TC concentration: 200 ppm, pH: 6.5). The experiments were performed under two different mixing modes: continuous stirring of the reactor contents at 400 rpm using a magnetic stirrer or immersion of the reactor in a 50 W ultrasonic bath. Prior to catalytic experiments, adsorption equilibrium was established under dark conditions for 60 min. Subsequently, UV light and/or sonication were initiated for the photocatalytic and sonophotocatalytic oxidation of TC.

### 4.3 Adsorption Study

Preliminary investigations of TC removal at various pH levels using a 30 mL solution sonicated under optimized conditions (catalyst concentration: 1000 ppm, initial TC concentration: 200 ppm, and up to 180 min), as presented in Figure 6a, confirmed that the optimal pH range should be 4–10. The results demonstrate maximum removal efficiency at pH 7, which can be partially attributed to enhanced Ce-MOF stability under neutral conditions. Figure 6b compares TC removal under sonication and simple stirring in a dark environment under standardized conditions (catalyst concentration: 1000 ppm, initial TC concentration: 200 ppm, pH 6.5). After 180 min, removal efficiencies reached 73.59% with sonication and 65.01% with stirring alone. The improved performance with ultrasound highlights its role in enhancing adsorption and mass transfer processes. Sonication increases turbulence, exposes fresh catalytic sites by breaking up particle agglomerates, and generates reactive oxygen species (ROS, e.g., •OH radicals) through acoustic cavitation, even in the absence of light.^80,81^ These results indicate that ultrasonic irradiation alone can promote TC removal via enhanced mixing, surface renewal, and ROS-mediated oxidation, independent of photodegradation.

**Figure 6:**
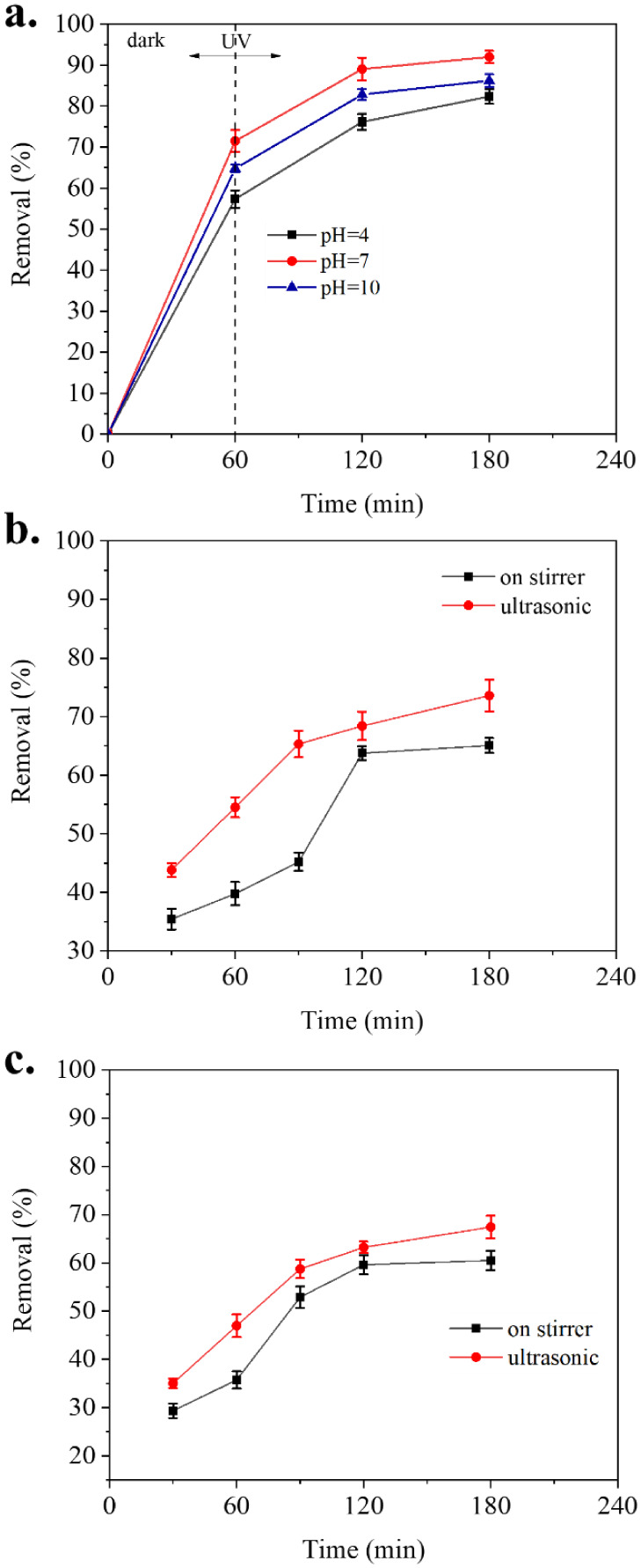
(a) Effect of pH on the removal efficiency of TC sonicated under UV irradiation. (b, c) Effect of using mechanical agitation (400 rpm) and sonication (50 W) on removal percentage in (b) a dark environment and (c) under UV irradiation. All experiments were carried out using 1000 ppm of catalyst and an initial TC concentration of 200 ppm. For (b) and (c), pH was 6.5.

To evaluate the combined effect of UV irradiation and sonication on TC removal, experiments were conducted under standardized conditions (catalyst concentration: 1000 ppm, initial TC concentration: 200 ppm, pH 6.5). As shown in Figure 6c, after 180 min, the removal efficiency reached 67.44% in the presence of both UV light and sonication, compared to 60.50% in the absence of sonication but with continuous stirring. The enhanced removal under UV demonstrates a synergistic interaction between adsorption promoted by efficient mixing and photocatalytic degradation induced by UV light. Stirring ensures uniform dispersion of the catalyst and facilitates mass transfer of TC molecules from the bulk solution to the catalyst surface, minimizing diffusion-limited effects. Sonication further enhances these processes via acoustic cavitation and microjet formation, which increase turbulence, deagglomerate the catalyst, renew active surface sites, and generate reactive oxygen species (e.g., •OH radicals), even in a dark environment. The combination of these effects explains the superior TC removal efficiency observed under UV and sonication conditions.^80–82^

In order to evaluate the Ce-MOF adsorption capacity, an experiment was conducted using a 30 mL solution under a 1000 ppm catalyst concentration, a 200 ppm initial TC concentration, and a pH of 6.5. The solution was stirred continuously under dark conditions for 24 h. Calculations using eqs 2 and 3 yielded a removal percentage of 96% and an adsorption capacity of 150 mg/g, respectively.

### 4.4 Photodegradation Mechanism

The band gap energy of the photocatalyst was determined to be 3.2 eV using the Tauc equation in our previous study, indicating promising photocatalytic capacity, as shown in Figure 7 upon UV irradiation. The primary species driving the degradation process are reactive intermediates such as superoxide radicals (·O_2_^–^), hydroxyl radicals (·OH), and positive holes (h^+^). A stronger suppression effect by a scavenger implies a greater involvement of the associated radical in the degradation reaction. As presented in previous work,^61^ 0.3 mM of EDTA, BQ, and IPA substantially inhibited the TC decomposition rate. The degree of inhibition by these scavengers followed the order: BQ > EDTA > IPA. The marked decrease in sonophotodegradation efficiency upon the addition of BQ and EDTA indicates that superoxide radicals (·O_2_^–^) play a predominant role. Conversely, the negligible effect observed with IPA suggests hydroxyl radicals (·OH) contribute minimally to TC breakdown. When Ce-MOF is exposed simultaneously to ultrasonic waves and light irradiation, electrons are excited from the valence band into the conduction band, leaving holes in their original position. The conduction band energy level of Ce-MOF is more negative than the O_2_/O_2_^–^ reduction potential, allowing photoexcited electrons to react with oxygen molecules and generate superoxide radicals (·O_2_^–^). Meanwhile, the valence band is positioned less positive than the oxidation potential for OH^–^ /·OH, facilitating the formation of hydroxyl radicals (·OH) via hole interactions with water molecules. The π–π conjugated structure inherent to the organic ligand of Ce-MOF enhances the rapid transfer of photoinduced electrons from holes to Ce sites, where they react with adsorbed TC molecules. On the Ce-MOF surface, the photogenerated charge carriers convert O_2_ and H_2_O into ·O_2_^–^ and ·OH radicals, respectively, which subsequently oxidize TC into CO_2_ and H_2_O. This overall pathway is summarized by the reaction numbered 4–16 ^61,83–85^

R1 $$Ce-MOF+h\nu \left(UV\right)\to Ce-MOF\left(h^{+}\mathrm{VB}\;\mathrm{and}\;e^{-}\mathrm{CB}\right)$$

R2 $$h^{+}+OH^{-}\to •OH$$

R3 $$h^{+}+H_{2}O+O_{2}\to •OH+H^{+}+•{O_{2}}^{-}$$

R4 $$O_{2}+e^{-}\to •{O_{2}}^{-}$$

R5 $${O_{2}}^{-}+H^{+}\to •H{O_{2}}^{-}$$

R6 $$2•H{O_{2}}^{-}\to O_{2}+H_{2}O_{2}$$

R7 $$H_{2}O_{2}+•{O_{2}}^{-}\to •OH+OH^{-}+O_{2}$$

R8 $$Ce-\mathrm{MOF}\left(h^{+}\mathrm{VB}\right),•OH,•{O_{2}}^{-},\cdot \cdot \cdot +\mathrm{TC}\to \mathrm{intermediates}\to CO_{2}+H_{2}O$$

R9 $$Ce-\mathrm{MOF}+\mathrm{hV}\to Ce-\mathrm{MOF}\left(e^{-}+h^{+}\right)$$

R10 $$O_{2}+e^{-}CB\to •O_{2}$$

R11 $$H_{2}O+h^{+}\mathrm{VB}\to •OH+H^{+}$$

R12 $${O_{2}}^{-}+TC\to CO_{2}+H_{2}O$$

R13 $$OH+TC\to CO_{2}+H_{2}O$$

**Figure 7:**
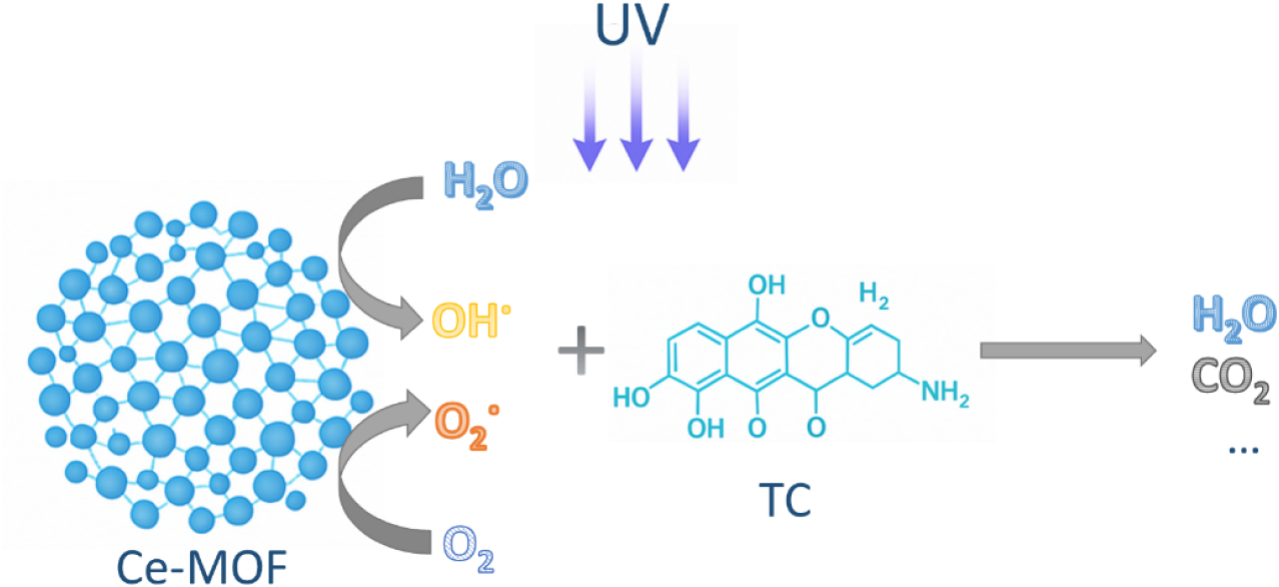
Photodegradation mechanism upon UV irradiation.

### 4.5 Design of TC Removal Experiments

#### 4.5.1 Provided Model by Software

The statistical modeling of important variables affecting TC removal efficiency via photocatalytic reaction was conducted. The investigated variables and their ranges are presented in Table 3. Table 4 provides the experimental design matrix following a central composite design and provides the measured removal efficiencies on each experiment. Analysis of variance (ANOVA) was employed to assess the model’s adequacy. The ANOVA results for the empirical second-order polynomial model in Table 5 yielded an F-value of 46.64, demonstrating high model accuracy.^86,87^ The model’s p-value of <0.0001 indicates statistical significance and a meaningful correlation with the experimental data, with only a 0.01% probability that the model’s F-value occurred by chance. The lack-of-fit F-value was 4.35. This nonsignificant lack of fit further validates the model’s predictive capability. Additionally, the predicted R^2^ (0.9073) shows reasonable agreement with the adjusted R^2^ (0.9461).

**Table 4:** Central Composite Design for Four Factors at Five Levels

Run	Coded TC concentration	Coded catalyst loading	Coded reaction Time	Coded pH	Experimental removal (%)
1	0	0	+2	0	90.24
2	0	+2	0	0	33.20
3	0	0	0	+2	20.32
4	0	–2	0	0	54.44
5	–1	+1	–1	+1	56.15
6	+1	+1	–1	–1	23.45
7	0	0	–2	0	61.54
8	–1	–1	–1	+1	57.86
9	+1	–1	+1	–1	50.01
10	0	0	0	0	85.22
11	+1	+1	+1	–1	43.00
12	0	0	0	0	85.60
13	0	0	0	–2	10.05
14	+1	+1	+1	+1	30.86
15	–1	–1	+1	–1	55.15
16	–2	0	0	0	60.24
17	+1	–1	+1	+1	55.20
18	–1	–1	–1	–1	40.70
19	+2	0	0	0	32.24
20	+1	+1	–1	+1	40.48
21	+1	–1	–1	+1	36.20
22	+1	–1	–1	–1	30.05
23	–1	–1	+1	+1	63.14
24	–1	+1	–1	–1	30.10
25	0	0	0	0	89.70
26	–1	+1	+1	+1	42.05
27	–1	+1	+1	–1	44.70

**Table 5:** Analysis of Variance (ANOVA) Test for Experimental Response (Removal Percentage) (A = Initial Concentration of TC; B = Catalyst Loading; C = Reaction Time; D = pH)

Source	Sum of Squares	df	Mean Square	F-value	p-value	
Model	11317.16	10	1131.72	46.64	<0.0001	significant
A	777.47	1	777.47	32.04	<0.0001	
B	599.93	1	599.93	24.72	0.0001	
C	667.04	1	667.04	27.49	<0.0001	
D	303.38	1	303.38	12.50	0.0027	
BC	145.52	1	145.52	6.00	0.0262	
CD	289.01	1	289.01	11.91	0.0033	
A^2^	2314.00	1	2314.00	95.36	<0.0001	
B^2^	2590.65	1	2590.65	106.76	<0.0001	
C^2^	192.30	1	192.30	7.92	0.0124	
D^2^	7050.31	1	7050.31	290.53	<0.0001	
Residual	388.27	16	24.27			
Lack of Fit	375.93	14	26.85	4.35	0.2024	not significant
Pure Error	12.34	2	6.17			
Cor. Total	11705.43	26				

Among the variables examined, several parameters demonstrated very high statistical significance (*p* < 0.001), indicating a strong and reliable influence on the response, as typically evaluated using ANOVA and regression analysis.^87^ These are the initial TC concentration (A), reaction time (C), and the second-order effects of initial TC concentration (A^2^), catalyst loading (B^2^), and pH (D^2^). Secondary significant parameters (*p* < 0.05), including catalyst loading (B) and pH (D), and the second-order effects of reaction time (C^2^) exhibited a measurable impact on the response,^87^ although less pronounced than the highly significant variables (*p* < 0.001). Furthermore, significant interactions were identified between certain factors (B–C and C–D). The empirical relationship between the significant input factors and the response is expressed in eq 17:

4 $$\mathrm{Total Removal}\;\left(\%\right)=\mathrm{-669.20123 + 2.47297 A + 0.134302 B + 1.02583 C + 121.15201 D - 0.000134 BC - 0.062964 CD}-{\mathrm{0.008502 A}}^{2}-{\mathrm{0.000044 B}}^{2}-{\mathrm{0.001483 C}}^{2}-{\mathrm{8.07964 D}}^{2}$$

Where total removal refers to combined contributions from adsorption, sonocatalytic degradation, photocatalytic degradation, and their synergies, A is the initial concentration of TC in ppm, B is the catalyst loading in ppm, C is the reaction time in min, and D is pH. The negative coefficients of the quadratic terms in the polynomial expression indicate their negative influence on photocatalytic performance.^88^

#### 4.5.2 Evaluation of Model Provided by DOE’s Method

The model demonstrates an excellent fit to the data, as evidenced by the high R^2^ value shown in Table 6. The close alignment between the adjusted and predicted R^2^ values with the regular R^2^ indicates the absence of significant overfitting, further confirming the model’s predictive capability. The adequate precision parameter, which measures the signal-to-noise ratio, substantially exceeds the minimum acceptable threshold of 4, providing additional validation of the model’s quality.

**Table 6:** Statistical Parameters Evaluating the Modeling of TC Removal Efficiency Based on Process Factors

R^2^	0.9668
Adjusted R^2^	0.9461
Predicted R^2^	0.9073
Adeq Precision	25.3883
SD	4.93
Mean	48.96
CV%	10.06

The residual analysis, presented in Figure 8a, plots residuals against the run number. The random distribution of points around the *x*-axis, without any discernible pattern, confirms the independence of model errors from the data sequence. Figure 8b depicts residuals versus predicted values, enabling verification of the constant variance assumption. The random scatter of residuals around the horizontal axis demonstrates that the variance remains independent of the process variable. Figure 8c presents the parity plot, which illustrates the strong agreement between the model predictions and the experimental measurements of TC removal efficiency.

**Figure 8:**
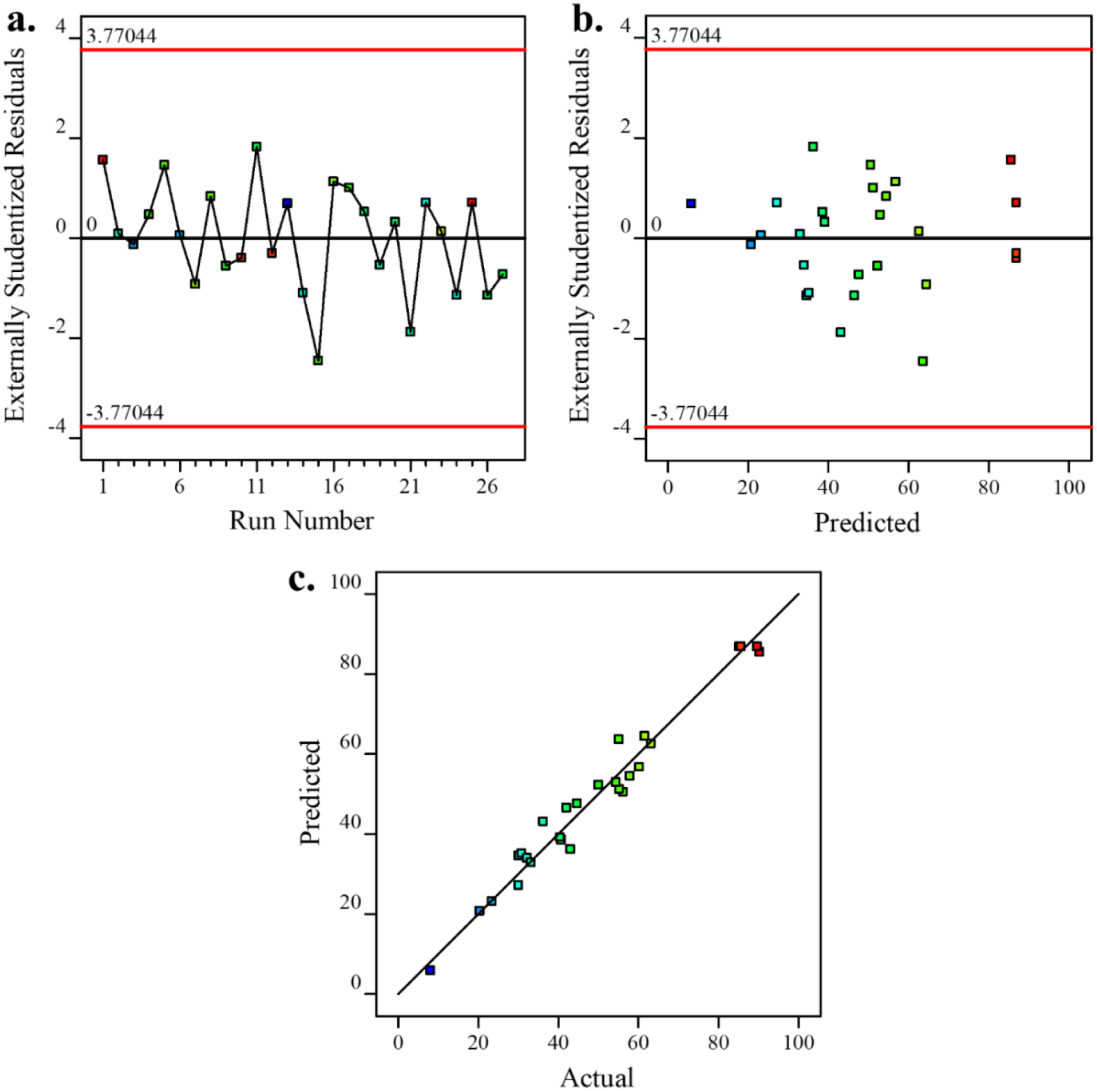
Analysis of residuals in statistical modeling of TC removal efficiency: (a) residuals vs run order; (b) residuals vs predicted; (c) parity plot of model predictions against experimental measurements.

#### 4.5.3 Effect of the Operating Parameters on the Photocatalytic Reaction

The relationship between TC concentration and removal rate exhibits a complex pattern, as illustrated in Figure 9a. Initially, increasing TC concentration enhances the removal rate; however, this effect diminishes as adsorption capacity reaches saturation. While increasing initial TC concentrations initially promotes both adsorption and degradation, continued increase in TC concentration led to reduced availability of active sites on the photocatalyst surface for TC molecules. This reduction in active sites decreases the probability of TC-catalyst interactions, thereby diminishing removal efficiency. The heightened competition among TC molecules for limited active sites results in a lower ratio of participating to nonparticipating TC molecules. Furthermore, excessive TC concentrations impair degradation through two mechanisms: first, the increased concentration causes TC to absorb much of the incident light, reducing photocatalyst activation and hydroxyl radical production; second, competition between TC, degradation intermediates, and commonly present anions (CO_3_^2–^,NO_2_^–^, Cl^–^, NO_3_^–^, SO_4_^2‑^ , PO_4_^3‑^, HCO_3_^–^) for adsorption on the Ce-MOF surface exacerbates inhibitory effects.^89,90^

**Figure 9:**
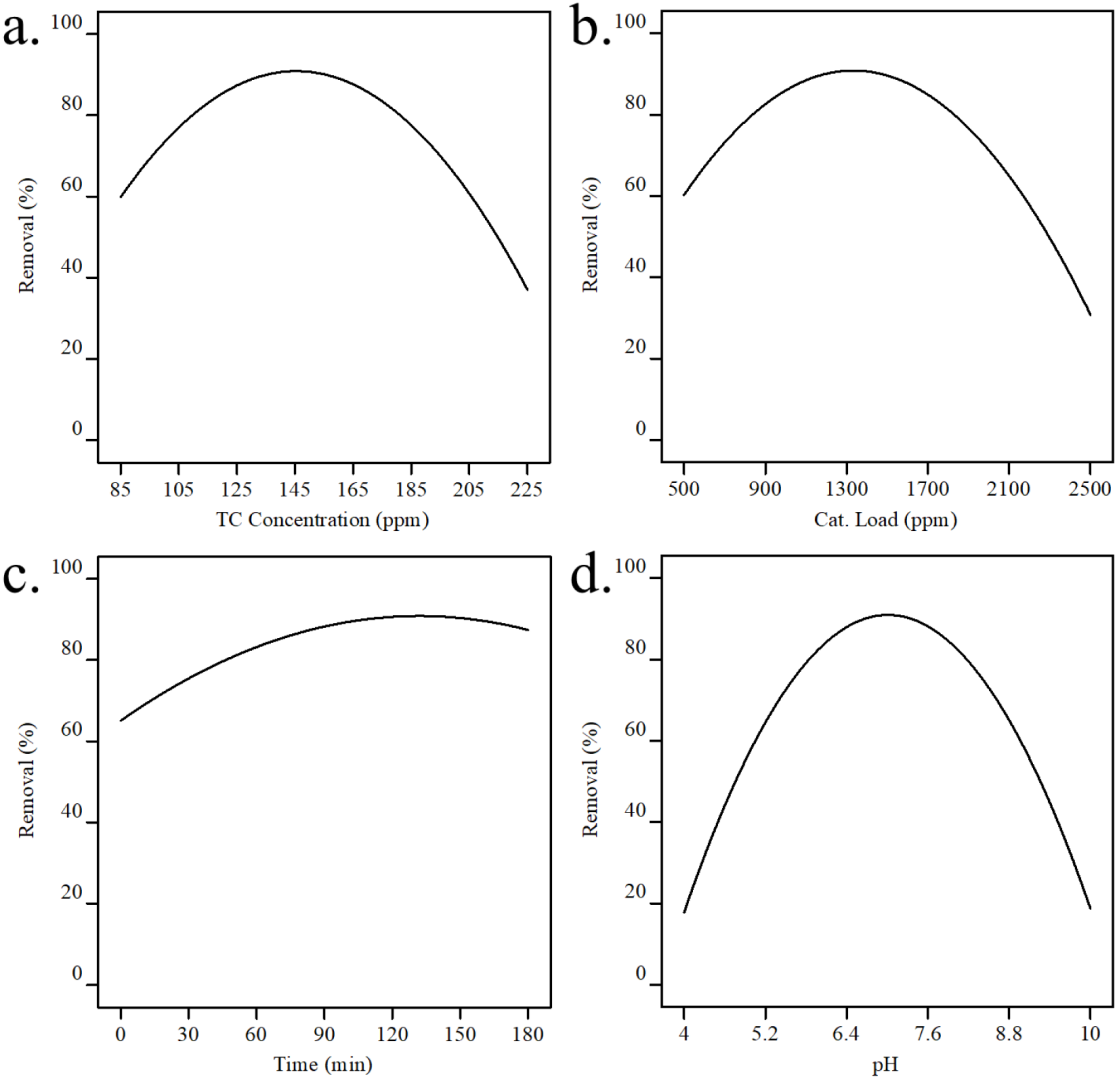
Single-factor analysis of tetracycline (TC) removal: (a) Effect of initial TC concentration on removal efficiency (Ce-MOF: 1500 ppm, reaction time: 90 min, pH: 7). (b) Effect of Ce-MOF concentration on removal efficiency (TC: 155 ppm, reaction time: 90 min, pH: 7). (c) Effect of sonophotocatalytic reaction time on removal efficiency (Ce-MOF: 1500 ppm, TC: 155 ppm, pH: 7). (d) Effect of initial pH on removal efficiency (Ce-MOF: 1500 ppm, TC: 155 ppm, reaction time: 90 min).

The economic viability of photocatalytic reactions depends significantly on optimizing photocatalyst usage. Higher photocatalyst concentrations generally improve removal efficiency by providing more active sites on the nanostructure surface, leading to an increased surface area and enhanced hydroxyl radical production. However, as demonstrated in Figure 9b, this improvement continues only until saturation occurs, after which the number of light-activated sites decreases.

The mechanism of photocatalysis involves light illumination of suspended photocatalysts, triggering electron transition from the valence band (VB) to the conduction band (CB), thereby generating electron–hole pairs. Figure 9c illustrates the effect of light exposure duration on TC degradation efficiency using Ce-MOF. The results demonstrate a direct correlation between the irradiation time and TC removal efficiency. The process begins with rapid TC removal due to increased adsorbent–adsorbate interactions at available active sites. As time progresses, greater dispersion of the adsorbent in the solution exposes more active sites to TC molecules until equilibrium is reached, when all active sites become occupied.

The pH dependence of TC removal is governed by the interplay between tetracycline speciation (pK_a1_ = 3.3, pK_a2_ = 7.8, pK_a3_ = 9.6) and the surface charge of Ce-MOF (isoelectric point pI ≈ 5).^52^ Below pH 3.3, TC exists as a cation (TCH_3_^+^), while the Ce-MOF surface is positively charged (pH < pI), resulting in electrostatic repulsion that limits adsorption. Between pH 3.3 and 7.8, TC adopts a zwitterionic form (TCH_2_^0^ /TCH^–^). In the range pH 5–7.8, the Ce-MOF surface becomes negatively charged (pH > pI), enabling moderate electrostatic attraction with the partially anionic TC species, complemented by attractive van der Waals forces and hydrogen bonding—leading to maximum removal at pH ≈ 7 (Figure 9d). Above pH 9.6, TC is fully anionic (TC^–^ /TC^2–^), and strong electrostatic repulsion with the negatively charged MOF surface, combined with OH^–^ competition for active sites and radical quenching, sharply reduces degradation efficiency.^91^ Moreover, extreme pH values (≤4 or ≥10) compromise Ce-MOF structural stability via protonation or hydroxylation of coordination bonds, which diminishes reactive oxygen species generation and active-site accessibility.^92^ Thus, the near-neutral pH optimum (≈7) emerges from a balanced synergy of (i) favorable electrostatic interactions, (ii) preserved catalyst integrity, and (iii) efficient photochemical oxidation—a mechanistic rationale that aligns with recent reports on pH-dependent pollutant-material interactions.^93^

#### 4.5.4 Optimization and Validation of Operating Parameters

The optimization of TC degradation conditions was performed using the desirability function approach. A numerical optimization software was employed to identify the optimal point that maximizes the desirability function. The software considers five possible goals—none, maximum, minimum, target, and within range—to construct desirability indices. All process variables (TC concentration, catalyst loading, reaction time, and pH) were assigned the goal “within range,” while the degradation percentage was set to “maximize.” The weight parameter, indicating relative importance, was used to emphasize upper or lower bounds or target values. To ensure reliability, validation studies were conducted in triplicate, and the experimental average values were compared with projected values to establish precise optimal conditions.

The numerical optimization process identified positions where the desirability function reached its maximum, determining precise optimal variables for TC removal. The selection of appropriate Ce-MOF conditions for photocatalytic degradation focused on maximizing TC removal efficiency under optimal conditions. Each goal was assigned a weight of 1 and an importance of 3, with all goals integrated into a unified desirability function incorporating multiple responses and variables. The software generated 100 formulations with a high desirability score of 1. The optimal formulation was selected using eq 18. Under the specified constraints and parameters, maximum TC degradation efficiency (92.24%) was achieved at pH 7.0, an initial TC concentration of 145 ppm, a reaction time of 111 min, and a catalyst loading of 1307 ppm.

5 $$\mathrm{Maximum}\equiv R{\left(\%\right)}^{*}\frac{I.C\left(\mathrm{ppm}\right)}{C_{\mathrm{catalyst}}{\left(\frac{g}{L}\right)}^{*}\mathrm{time}}$$

where R represents removal efficiency, I.C. denotes initial TC concentration, and C_catalyst_ represents catalyst loading.

To verify the model’s predictive accuracy for maximum TC degradation percentage, validation experiments were conducted under optimal conditions. Three replicate experiments yielded TC removal efficiencies of 89.48%, 88.53%, and 88.29%. The mean squared error (MSE), calculated using eq 19:

6 $$\mathrm{MSE}=\frac{1}{n}\overset{n}{\underset{i=1}{\sum }}{\left(y_{\mathrm{mi}}-y_{\mathrm{ei}}\right)}^{2}$$

where y_mi_ represents the experimental actual value, y_ei_ denotes the software-predicted value (optimal point in %), and n indicates the number of experimental repetitions. The calculated MSE was less than 4 confirming appropriate agreement between predicted and experimental values and validating the model’s ability to simulate TC photocatalytic degradation. Further optimization investigations at higher pH (7.5) produced an average maximum degradation of 88.17% across three replicates, while extended reaction time (180 min) resulted in an average maximum degradation of 92.06%. These results confirm the practical utility of the software-recommended optimal conditions.

#### 4.5.5 Investigating the Effect of the Interaction between the Parameters

Another significant advantage of experimental design over traditional methods is its ability to evaluate parameter interactions and their impact on the final response. These interactions occur when the effect of one parameter depends on the level of another parameter, influencing the final model. Three-dimensional surface plots serve as graphical representations of the regression equation, facilitating the determination of optimal variable values and providing an in-depth understanding of variable interactions within the considered range.^94,95^ Figure 10 presents the interactions between the four independent variables (catalyst load, reaction time, initial concentration, and pH) and their effect on TC removal efficiency.

**Figure 10:**
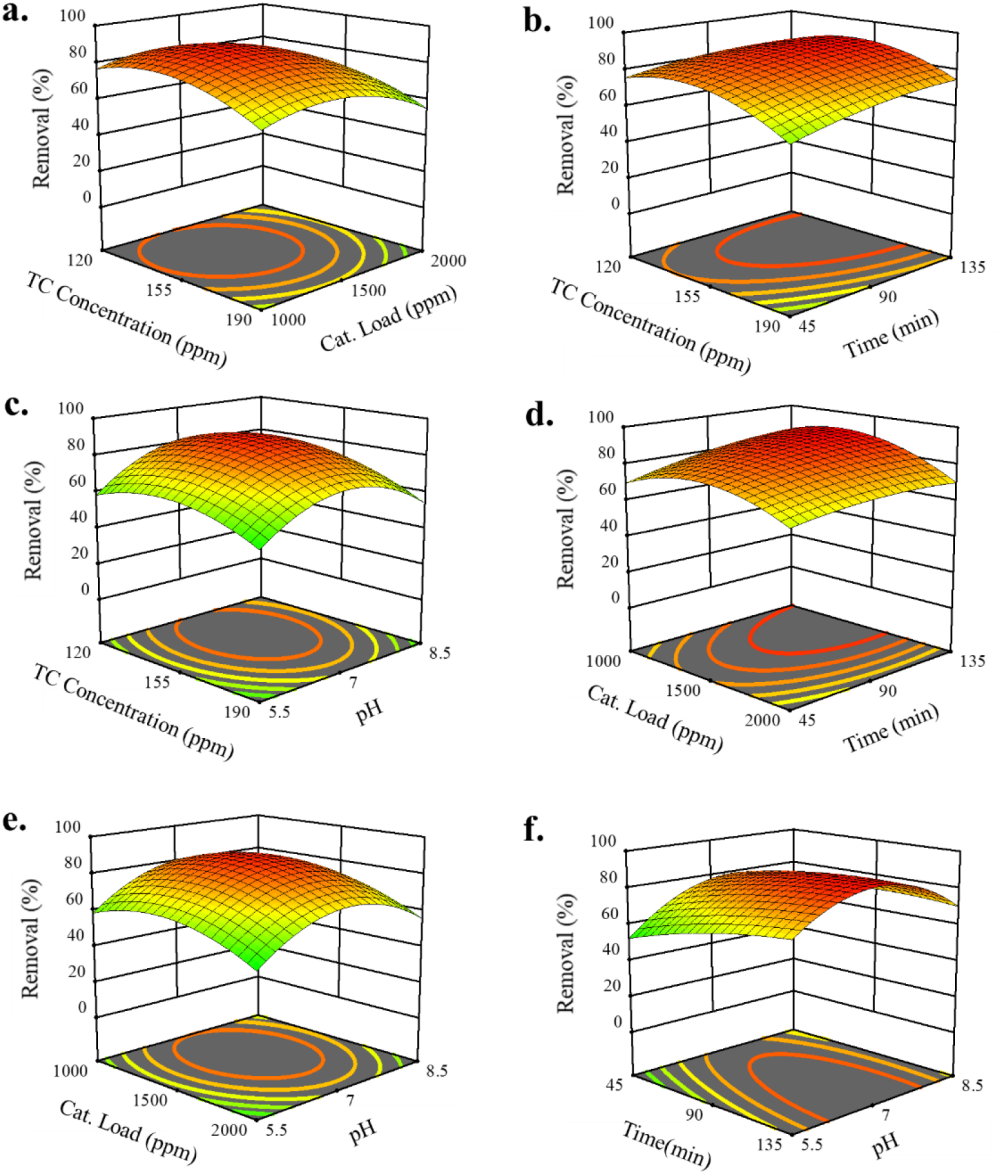
(a) Effect of TC concentration and catalyst loading (time = 90 min and pH = 7). (b) Effect of TC concentration and reaction time (catalyst loading = 1500 ppm and pH = 7). (c) Effect of TC concentration and pH (catalyst loading = 1500 ppm and reaction time = 90 min). (d) Effect of catalyst loading and reaction time (TC concentration = 155 ppm and pH = 7). (e) Effect of catalyst loading and pH (time = 90 min and TC concentration = 155 ppm). (f) Effect of reaction time and pH (TC concentration = 155 ppm and catalyst loading = 1500 ppm) on removal percentage.

According to Figure 10a, the removal efficiency decreased with an increasing initial TC concentration but improved at higher catalyst loading. In fact, higher pollutant concentrations saturate the active sites of Ce-MOF and attenuate degradation, whereas increasing the catalyst amount provides more reactive sites for photocatalytic reactions. The optimal removal was observed at lower to moderate TC concentrations combined with moderate to high catalyst load, confirming that active site availability is a key factor in determining efficiency.^96,97^ As shown in Figure 10b, longer reaction times significantly enhance TC removal, while higher initial concentrations reduce it. Extended UV irradiation allows for more reactive species to form, promoting degradation over time. In contrast, high pollutant loads lead to competition for limited active sites, which slows the overall removal process. The best results were obtained with moderate to low TC concentrations under prolonged irradiation, demonstrating the importance of balancing pollutant load and exposure time.^98,99^

It can be seen from Figure 10c that the highest removal efficiency was observed at neutral pH (∼6–7), with a decline under strongly acidic or basic conditions. Extreme pH can alter the surface charge of Ce-MOF and affect protonation/deprotonation equilibria, reducing the formation of reactive species and overall photocatalytic activity. Lower TC concentrations further improved removal, indicating that neutral pH and moderate pollutant levels create the most favorable conditions for degradation.^52,100^ In Figure 10d, removal efficiency increased with both higher catalyst load and longer reaction time. More catalyst load increases the number of available active sites, and prolonged irradiation allows sustained generation of reactive species, resulting in more efficient TC degradation. The optimal region was found at long reaction times with high catalyst loading.^97,99^

As shown in Figure 10e, increasing catalyst loading improved removal efficiency, particularly at neutral pH. A higher catalyst amount provides more active sites for TC adsorption and reaction, while extreme pH conditions may destabilize the Ce-MOF structure or inhibit surface reactions. Therefore, high catalyst loading combined with neutral pH yielded the most effective photocatalytic performance.^97,101,102^ It is shown in Figure 10f that neutral pH and longer reaction times promote TC degradation. Actually, pH values above 7 result in decreased degradation efficiency. This behavior can be explained by the photocatalyst’s surface charge. According to zeta potential analysis, the Ce-MOF surface maintains a positive charge in acidic media (up to pH ∼ 5). As solution pH increases, the number of negatively charged sites increases, enhancing TC cation adsorption through electrostatic attraction. In basic conditions, TC begins to form anionic species, with their proportion increasing at higher pH values.^103–105^ Consequently, electrostatic repulsion develops between TC anions and the negatively charged Ce-MOF surface. Reaction times exceeding 111 min have minimal impact on TC degradation.

Overall, among all factors, catalyst load and reaction time had the strongest positive impact on removal efficiency. Higher initial TC concentrations decreased efficiency due to limited active sites, while pH effects were most favorable near neutrality. The optimal conditions for maximum removal of TC were, therefore, neutral pH (∼6–7), high catalyst loading, longer reaction time, and moderate to low initial TC concentration. These results highlight that photocatalytic efficiency is governed by the balance between active site availability, sufficient UV exposure, pollutant concentration, and pH-dependent surface chemistry. The model proves that pH-reaction time and catalyst loading-reaction time interactions adversely impact TC degradation.

### 4.6 Sonophotocatalytic Degradation under Visible Light

The band gap energy obtained through DRS analysis suggests potential photocatalytic activity in the visible light spectrum.^106^ To evaluate the TC removal efficiency under visible light conditions (30 W SNR LED), experiments were conducted using a solution prepared under optimal conditions. The solution was subjected to a sonophotocatalytic treatment. The TC removal efficiency reached 68% after 180 min of visible light exposure.

### 4.7 Stability and Repeatability of Ce-MOF

The practical viability of catalysts depends heavily on their stability and reusability. To assess these characteristics, the Ce-MOF photocatalyst underwent multiple consecutive experimental runs under conditions identical to those used for the fresh catalyst. Between runs, a thorough recovery protocol was implemented: the Ce-MOF photocatalyst was collected after each cycle, washed extensively with water and ethanol, and dried at 60 °C for 24 h before subsequent use. This recovery and reuse process was repeated through six complete cycles, with all experiments conducted under previously established optimal conditions to ensure consistent evaluation.

The results demonstrate the economic reusability of the photocatalyst while revealing a gradual decline in performance across successive cycles. As shown in Figure 11, the initial TC removal efficiency under the optimal conditions reached 89.48% in the first cycle. Performance in subsequent cycles showed a progressive decrease, with removal efficiencies of 88.53%, 82.91%, 78.16%, 67.32%, and 61.59% for the second through the sixth cycles, respectively.

**Figure 11:**
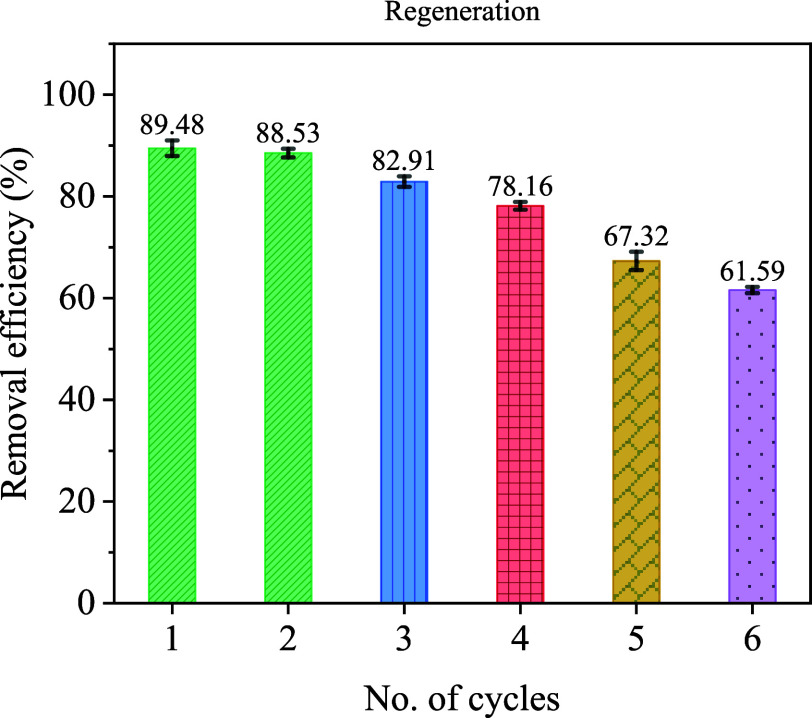
Removal efficiency of Ce-MOF at different cycle numbers.

The observed decline in activity after six cycles is not due to structural degradation of the Ce-MOF. As seen in Figure 11, the efficiency drop from cycle 1 to cycle 3 was not severe, indicating that the washing mechanism was effective and fouling did not play an important role. As discussed in Sections 4.1.1 (FTIR analysis) and 4.1.2 (XRD analysis), and illustrated in Figures 2a and 3, the structure of Ce-MOF remains unchanged, with no phase change or loss of crystallinity—a behavior consistent with robust MOFs that withstand ultrasonic irradiation (e.g., UiO-66 series under moderate sonication).^107,108^ While the XRD patterns in Figure 3 confirm no phase change, the modest reduction in XRD peak intensities after recycling indicates partial amorphization of the Ce-MOF framework. Importantly, for the initial cycles (1–3), where efficiency remains high (>80%), this crystallinity loss does not compromise catalytic performance, suggesting that active sites are preserved and the increased defect density may even contribute to adsorption capacity.^109^ However, the sharp decline from cycle 4 to cycle 6 is driven by the cumulative effects of agglomeration and minor Ce leaching, with amorphization playing a secondary, exacerbating role by creating more defect sites that facilitate particle–particle adhesion. This leaching could potentially be mitigated by optimizing the washing protocol.

Instead, the primary cause is surface agglomeration of MOF particles induced by ultrasonic cavitation, which progressively reduces the accessible active surface area—a common phenomenon in nanomaterial-based sonocatalysts.^110^ A minor contribution comes from limited Ce leaching, which does not compromise the structural integrity.

Moreover, this gradual reduction in photocatalytic activity can be attributed to two primary factors: photocorrosion, which presents a significant challenge to Ce-MOF photocatalyst performance, and surface saturation resulting from the accumulation of undegraded TC molecules and photocatalytic reaction products within the Ce-MOF structure.^61,111^ Thus, the material exhibits practical stability, and the gradual activity drop is a reversible physical surface phenomenon rather than a permanent structural collapse.

Comparing the performance of Ce-MOF with those of CoP-3^31^ and Zn@Co–N–C-1000/PMS^14^ MOF, it can be concluded that the Ce-MOF is challenged with higher TC concentrations (85–225 ppm). Moreover, degradation has been achieved across a wide range of pH 4–10 without significant changes in TC removal efficiency. It also demonstrates stable reusability, retaining over 70% activity across four consecutive cycles and over 60% upon six cyclese5f*a performance that surpasses the stability reported for CoP-3.

## 5 Conclusions

This research demonstrates the effective application of a Ce-MOF as a photocatalyst for TC removal from aqueous solutions. Through systematic optimization using Design Expert software, the study established optimal conditions for TC removal: 89% removal efficiency was achieved with a 1307 ppm catalyst concentration treating a 145 ppm TC solution at pH 7.0 over 111 min. This exceeds our previous results of 82% under similar conditions. The study revealed that photocatalytic activity demonstrates a nonlinear relationship with operational parameters, increasing with initial TC concentration, catalyst dosage, reaction time, and pH until reaching optimal values. The catalyst exhibited sustained performance through six consecutive cycles, underscoring its practical viability for water treatment applications. The Ce-MOF photocatalyst’s stability and cost-effectiveness position it as a promising solution for organic pollutant remediation in water treatment applications.

Sonochemical synthesis emerged as the preferred preparation method, with ultrasonic bath radiation proving superior to conventional stirring for treatment efficacy. Several promising directions emerge for future research to advance this technology toward practical implementation. Priority areas include evaluating the photocatalyst’s performance in real wastewater from hospital effluent streams, developing fixed-substrate configurations to enhance separation efficiency, and investigating temperature effects on removal efficiency. Additional research opportunities encompass scale-up studies for industrial applications, expansion to other pharmaceutical and organic pollutants, optimization of UV radiation intensity, and exploration of alternative catalyst recovery methods.
